# DNA deletion as a mechanism for developmentally programmed centromere loss

**DOI:** 10.1093/nar/gkv1110

**Published:** 2015-10-25

**Authors:** Maoussi Lhuillier-Akakpo, Frédéric Guérin, Andrea Frapporti, Sandra Duharcourt

**Affiliations:** Institut Jacques Monod, CNRS, UMR 7592, Université Paris Diderot, Sorbonne Paris Cité, Paris, F-75205 France

## Abstract

A hallmark of active centromeres is the presence of the histone H3 variant CenH3 in the centromeric chromatin, which ensures faithful genome distribution at each cell division. A functional centromere can be inactivated, but the molecular mechanisms underlying the process of centromere inactivation remain largely unknown. Here, we describe the loss of CenH3 protein as part of a developmental program leading to the formation of the somatic nucleus in the eukaryote *Paramecium*. We identify two proteins whose depletion prevents developmental loss of CenH3: the domesticated transposase Pgm involved in the formation of DNA double strand cleavages and the Polycomb-like lysine methyltransferase Ezl1 necessary for trimethylation of histone H3 on lysine 9 and lysine 27. Taken together, our data support a model in which developmentally programmed centromere loss is caused by the elimination of DNA sequences associated with CenH3.

## INTRODUCTION

The centromere is the specialized chromosomal region that defines the assembly sites for the kinetochore and is therefore essential for faithful genome distribution at each cell division. In most eukaryotes, functional centromeres are marked by the presence of the histone H3 variant CenH3 that replaces canonical H3 in centromeric nucleosomes. CenH3 is thought to serve as a loading platform for the recruitment of other kinetochore proteins (1). In general, chromosomes contain a single region where centromere DNA sequences assemble kinetochores. However, genome rearrangements can lead to the accidental emergence of an additional centromere on the same chromosome. Such dicentric chromosomes are generally unstable during mitosis (2,3), but can give rise to functionally monocentric chromosomes that segregate normally during cell division, when one centromere is inactivated (4). Inactivated centromeres are characterized by the absence of primary constriction on metaphase chromosomes and key kinetochore proteins (5,6). The molecular mechanisms underlying the process of centromere inactivation are still poorly understood, even though several studies suggested two possible pathways: (i) deletion of centromere DNA sequences and (ii) epigenetic inactivation when centromeric DNA is retained but CenH3 is absent (7–11).

**Figure 1.**
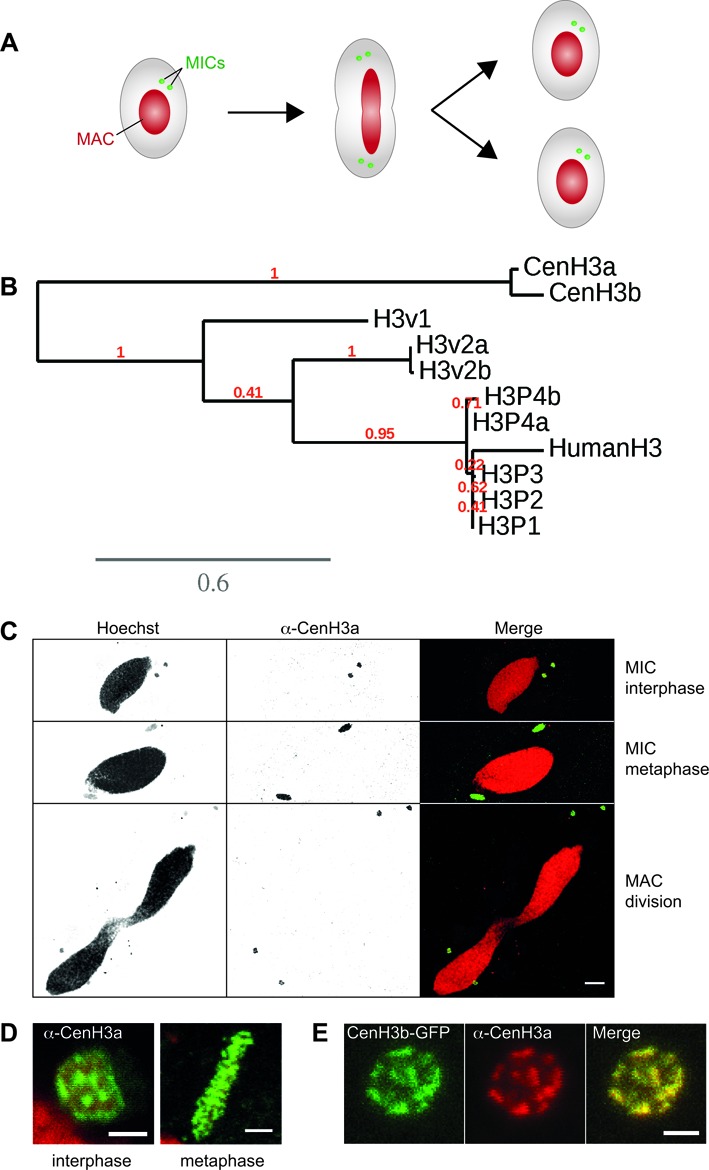
The *Paramecium tetraurelia* centromeric histone H3 variant. (**A**) Schematic representation of key nuclear events during *Paramecium* cell division. MAC: macronucleus; MICs: micronuclei. Note that the MICs divide before the MAC. (**B**) Phylogenetic analysis of *P. tetraurelia* H3 and H3 variants proteins. H3 proteins were retrieved using BLAST (55). Duplicates from the last whole genome duplication are named a and b. Multiple alignments were performed with the MUSCLE software (56). Phylogenetic analysis was carried out using PhyML 3.0 (bootstrapping procedure, 100 bootstraps) with default parameters and trees were visualized using TreeDyn (57). A scale bar in expected substitutions per site is provided for branch length. See also Supplementary Figures S1 and S2. (**C**) Immunostaining with CenH3a antibody at different stages of the cell cycle. Scale bar is 10 μm. (**D**) Magnified views of the MICs during interphase and metaphase. Scale bar is 2 μm. See also Supplementary Figure S3. (**E**) Colocalization of CenH3a and CenH3b proteins in the MICs during interphase. Immunostaining with CenH3a antibody of *CENH3b-GFP* transformed cells during vegetative growth. Scale bar is 2 μm.

The eukaryote *Paramecium tetraurelia* provides an interesting context to dissect the general mechanisms that control centromere inactivation. In this unicellular organism, two types of nuclei with distinct modes of chromosome segregation coexist in the same cytoplasm (Figure 1A). The highly polyploid somatic macronucleus (MAC) is responsible for gene expression, while the diploid germline micronuclei (MICs) ensure the transmission of the genetic material to the next sexual generation. The two nuclei divide at each cell division during vegetative growth, but only the MIC chromosomes segregate in daughter cells through conventional mitosis (12) (Figure 1A). In contrast to MICs, MAC undergoes nuclear division through a nonmitotic process that does not seem to involve chromosome condensation or mitotic spindle assembly (13,14). Yet both MIC and MAC develop from the zygotic nucleus formed after meiosis of the MICs. Mitotic divisions of the zygotic nucleus produce four identical diploid nuclei that differentiate into new MICs and new MACs, while the maternal MAC is progressively destroyed. Development of the somatic MAC from the zygotic nucleus is characterized by extensive and reproducible remodeling of the genome, which includes the precise excision of numerous, short, unique noncoding Internal Eliminated Sequences (IESs) and the elimination of about 25 Mb of MIC-limited regions, often containing repetitive sequences (15). As a result, *P. tetraurelia* germline MIC chromosomes—their sequence and precise number are not yet known—are fragmented into approximately 200 shorter MAC molecules healed by *de novo* telomere addition (16). Key proteins required for programmed DNA elimination are: (i) the putative endonuclease PiggyMac (Pgm), necessary for the introduction of DNA double strand breaks at the extremities of IESs (17,18); (ii) the Polycomb-like putative histone methyltransferase Ezl1, necessary for histone H3 trimethylation of lysine 9 and lysine 27 during macronuclear development (19); (iii) the Dicer-like proteins 2 and 3 (Dcl2 and Dcl3), necessary for the biogenesis of 25 nt scanRNAs involved in the maternal control of DNA elimination (20); (iv) the Dicer-like 5 protein (Dcl5), necessary for the production of 26–30 nt iesRNAs (21).

Here, we identify the centromeric histone variant CenH3 of *P. tetraurelia*. We show that the centromeric function is restricted to the germline MICs and that the CenH3 protein is absent from the somatic MAC. We also show that the CenH3 protein is lost during somatic differentiation, in the same developmental time window as DNA elimination events. We further demonstrate that CenH3 loss requires key proteins involved in programmed DNA elimination. Taken together, our data support a model in which developmentally programmed centromere loss is caused by the physical elimination of DNA sequences associated with CenH3.

## MATERIALS AND METHODS

### *Paramecium* strains and cultivation

All experiments were carried out with the entirely homozygous strain 51 of *P. tetraurelia*. Cells were grown in wheat grass powder (WGP) (Pines International) infusion medium bacterized the day before use with *Klebsiella pneumoniae*, unless otherwise stated, and supplemented with 0.8 mg/ml β-sitosterol (Merck). Cultivation, autogamy and conjugation were carried out at 27°C as described (22,23).

### CenH3a antibody and indirect immunofluorescence

Polyclonal rabbit antibodies were raised to an amino-terminal peptide sequence (positions 4–23: KTTKENNNQSFQVDNNEKMP) of the CenH3a protein with Quality Controlled Biochemicals. Although the affinity-purified CenH3a antibodies do not work in Western blot, we demonstrated their specificity by dot blot and competition assays (Supplementary Figure S4A–B) with secondary horseradish peroxidase-conjugated donkey anti-rabbit IgG antibody (Promega), followed by detection by ECL (SuperSignal West Pico Chemiluminescent Substrate, Thermo Scientific). For immunostaining, two protocols were used and gave the same results. Cells were treated as described in (19) or cells were permeabilized for 5 min in PHEM (60 mM PIPES, 25 mM HEPES, 10 mM EGTA, 2 mM MgCl2, pH 6.9) with 1% Triton (Sigma-Aldrich), then fixed for 30 min in PHEM with 1.3% formaldehyde (Sigma-Aldrich). Cells were washed in TBST (10 mM Tris pH 7.4, 150 mM NaCl, 1% Tween 20, 10 mM EGTA, 2 mM MgCl2) with 3% BSA (Sigma-Aldrich) and incubated with CenH3a antibody (dilution 1:500), washed, incubated with secondary anti-rabbit antibody (Alexa Fluor A488 or A568, dilution 1:500)(Life Technologies), stained with 0.4 μg/ml Hoechst (Sigma-Aldrich) and mounted on microscope slides in Citifluor AF2 glycerol solution (Biovalley). Images were acquired using laser-scanning confocal microscopes (Leica DMI 6000 or ZEISS LSM 710) and a Plan-Apochromat 63×/1.40 oil DIC M27 objective. Z-series were performed with Z-steps of 0.5 μm.

### Injection of GFP fusion transgenes, GFP localization and fluorescence quantification

For the construction of in-frame CenH3 fusion, the coding sequence of the *CENH3a* and *CENH3b* genes were inserted into the plasmid pTI (Baptiste Saudemont and Eric Meyer, unpublished) upstream of a GFP coding fragment adapted to *Paramecium* codon usage. As a result, the GFP is fused to the C-terminus of CenH3a and CenH3b and the fusion protein is expressed under the control of the constitutive promoter of the Elongation Factor Tu (Supplementary Figure S3A). A flexible linker sequence was added between the *CENH3* and the GFP coding sequences (Supplementary Figure S3A). Plasmids carrying the *CENH3a*- and *CENH3b*- GFP fusion transgenes were linearized by *BglI* and were microinjected into the MAC of vegetative 51 cells. For localization of CenH3a-GFP and CenH3b-GFP proteins, cells transformed with GFP transgenes were fixed as described in (19). Quantification of GFP signal intensity was performed with the ImageJ software. The average fluorescence intensities of CenH3b-GFP in the two MICs (signal) and in corresponding volumes of the cytoplasm (background) were measured. The mean and standard deviation of the corrected average fluorescence intensities values (signal minus background) were calculated using at least 30 individual cells for each silencing condition.

### Gene silencing experiments

Plasmids used for dsRNA production in silencing experiments were obtained by cloning PCR products from each gene using plasmid L4440 and *Escherichia coli* strain HT115 DE3, as previously described (24). To maximize silencing specificity, dsRNA sequences corresponding to the most divergent region of *CENH3* genes were chosen (77% identity at the nucleotide level and no 22 pb segment of perfect identity): 7–174 and 7–186 of PTETG46600001001 (*CENH3a*) and PTETG6500002001 (*CENH3b*), respectively. The fragments used for *ND7*, *ICL7a*, *DCL2*, *DCL3*, *DCL5*, *EZL1-1* and *PGM-1* are those previously described (19).

Silencing media were prepared by inoculating precultures of the appropriate bacterial strains into WGP medium containing 0.1 mg/ml ampicillin (Sigma-Aldrich). Following 6–8 h of shaking at 37°C, bacterial cultures were diluted into the same medium to OD_600_ = 0.08 and supplemented with 0.4 mM IPTG (Euromedex) to induce the synthesis of dsRNA. Following overnight induction at 37°C, 0.8 mg/ml of β-sitosterol (Merck) were added before use. *Paramecium tetraurelia* cells were first grown in standard *K. pneumoniae* medium for 20–30 vegetative fissions then washed twice in silencing medium. Cells were grown for eight to ten additional vegetative fissions in silencing medium (freshly induced medium was added the second day) before starvation-induced autogamy. Progression of autogamy was monitored by Hoechst (Sigma-Aldrich) staining, and cells were generally 100% autogamous at day 1 of starvation. At day 3 or 4, 30–60 autogamous cells were picked and transferred individually to 200 μl of *K. pneumoniae* medium to monitor growth of sexual progeny.

## RESULTS

### Identification of centromeric histone H3 variants

In order to identify the centromeric histone CenH3 of *P. tetraurelia*, we first searched for histone H3 homologues in the MAC genome assembly (25,26). Indeed, CenH3 proteins bear strong homology to canonical histone H3 protein but contain distinct sequence features, including a noncanonical N-terminal tail, a more divergent core histone fold and a slightly longer loop 1 region (27). We identified 16 genes encoding ten different histone H3 proteins (Supplementary Figures S1 and Figure 1B). The exceptionally high number of H3 variants found in the *Paramecium* genome is due in part to the presence of closely related genes that arose from a recent whole genome duplication (16).

Among the ten identified histone H3 proteins, five (H3P1, H3P2, H3P3, H3P4a, H3P4b) are closely related to the human canonical histone H3 (Figure 1B). Both H3P4 proteins have an isoleucine at position 89, a signature for the transcription-associated H3.3 variant (28), instead of a valine in the other three canonical H3 proteins (H3P1, H3P2, H3P3) (Supplementary Figure S2). Therefore H3P4 proteins are most likely H3.3 variants. In contrast to other organisms, *Paramecium* canonical H3 proteins and putative H3.3 variants are quite divergent from the highly conserved histone H3 (81% overall identity and 90% similarity with human H3). The differences include the addition of a few amino acids in the N-terminal tail and several amino acid substitutions throughout the proteins (Supplementary Figure S2). Consistent with previous reports (29–32), ciliate histone proteins appear to evolve more rapidly.

The other five histone H3 variants are much more divergent (Figure 1B). Remarkably, the H3v1 and H3v2 proteins harbor deletions and/or substitutions around critical lysine residues (K9, K27, K79) usually associated with post-translational modifications (Supplementary Figure S2). CenH3a and CenH3b can be distinguished from canonical H3 and other H3 histone variants by their distinct features. The histone fold domains of CenH3a and CenH3b are only 44% identical to other histone H3 variants in *P. tetraurelia*. These two proteins harbor a longer loop 1 region within the histone fold domain, a highly divergent N-terminal tail and an extension of the C-terminal tail (Supplementary Figure S2). Thus, CenH3a and CenH3b present the hallmarks of CenH3 proteins (27). Compared to the other H3 proteins, CenH3a and CenH3b variants are the most divergent, and are constitutively expressed at low levels during the life cycle (Supplementary Figure S1). Even though they are duplicates from the last whole genome duplication, the proteins only share 90% overall amino acid identity and are most divergent in the N-terminal tail (76% identity).

### CenH3 proteins localize exclusively to the germline MICs

C-terminal GFP fusions were used to analyze the subcellular localization of the CenH3a and CenH3b proteins (see Materials and Methods and Supplementary Figure S3A). Transgenes expressing each fusion protein were microinjected into the MAC of vegetative cells and the resulting transformants were grown (Supplementary Figure S3A). At each cell division during vegetative growth, the micro- and macronuclei divide (Figure 1A). In cells expressing CenH3a- or CenH3b- GFP fusion proteins, GFP fluorescence was never detected in the MAC, but was exclusively found in the MICs of the transformed clones (Supplementary Figure S3B–E). To confirm these observations, we performed indirect immunostaining experiments with a polyclonal antibody that was shown to be highly specific for CenH3a (see Materials and Methods and Supplementary Figure S4A–C). As for the GFP fusion proteins (Supplementary Figure S3B–E), the endogenous CenH3a protein was found in the MICs at all stages of the cell cycle (Figure 1C and D) and was completely absent from the MAC (Figure 1C). During interphase, CenH3 staining forms large nuclear dots (Figure 1D, Supplementary Figure S3 panels C and E), suggesting that centromeres cluster as described in other systems (33,34). The CenH3a and CenH3b proteins colocalize at subnuclear regions in the MICs, as shown using CenH3b-GFP and the CenH3a antibody (Figure 1E). This punctate staining relocated on the metaphase plate (Figure 1D, Supplementary Figure S3 panels C and E). Previous studies had estimated the number of MIC chromosomes to be between 30 and 60 pairs depending on *Paramecium* species (12,35–36). Due to the small size and large number of MIC chromosomes, we could not count them with accuracy, nor could we determine whether MIC chromosomes are monocentric or holocentric, i.e centromeres dispersed all along the chromosome, as in *C. elegans* (37).

### CenH3a is required for correct micronuclear division at mitosis

**Figure 2.**
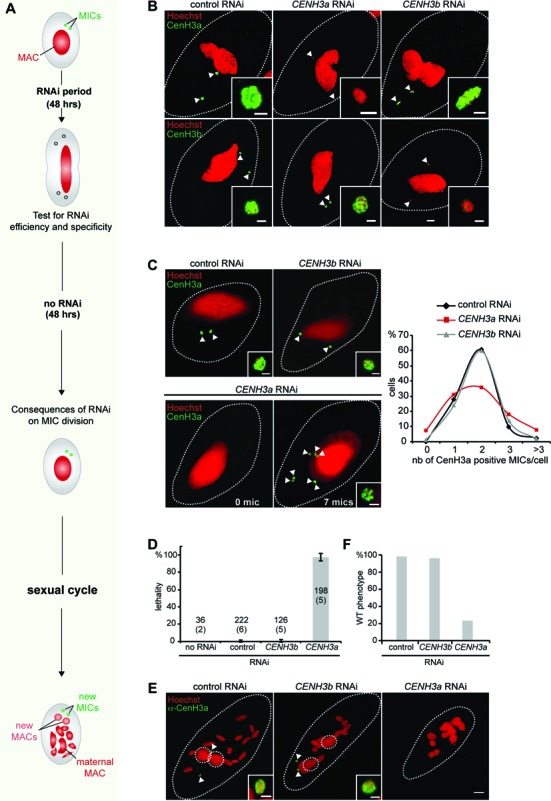
Functional analysis of *CENH3* genes. (**A**) Schematic representation of the experimental design. (**B**) Efficient and specific silencing of *CENH3* genes was assayed at the protein level. Upper panel: immunostaining with CenH3a antibody after control (*ND7*), *CENH3a* or *CENH3b* RNAi. Lower panel: GFP detection in *CENH3b*-*GFP* transformed cells after control (*ND7*), *CENH3a* or *CENH3b* silencing. Scale bar is 10 μm. Magnified views of the MICs (white arrow) are shown. Scale bar is 2 μm. See also Supplementary Figure S4. (**C**) Consequences of *CENH3* gene silencing were analyzed by CenH3a immunostaining 48 h after RNAi release. RNAi conditions are indicated above each image. Magnified views of the MICs (white arrow) are shown. Scale bar is 2 μm. The linear chart shows quantification of the number of MICs per cell (determined by immunostaining with CenH3a antibody). More than 100 cells were scored in each condition. (**D**) Lethality of sexual progeny following silencing of *CENH3* genes during vegetative growth. The gene targeted in each silencing experiment is indicated. The *ND7* gene was used as control, since its silencing has no effect on sexual processes. The sexual process was also performed in standard *K. pneumoniae* medium (no RNAi). Cells were starved in each medium to induce sexual events and, following 3–4 days of starvation, cells were transferred individually to *K. pneumoniae* medium to monitor growth of sexual progeny. The total number of cells analyzed for each RNAi and the number of independent experiments (in parenthesis) are indicated. Error bars indicate the standard deviation for each condition. Of note, no lethality (97% of viable progeny) was observed in the post-autogamous progeny of *CENH3b-GFP* transformed cells in the experiment presented in panels B and C, indicating that expression of *GFP* fusion did not interfere with normal progression of autogamy. (**E**) Cytological defects following silencing of *CENH3* genes during vegetative growth were monitored, by immunostaining with CenH3a antibody, on cells at the end point of sexual cycle, following 3–4 days of starvation. Dashed white circles indicate the new MACs and white arrows the new MICs. The other Hoechst-stained nuclei are fragments from the maternal MAC. Scale bar is 10 μm. Magnified views of one MIC are presented. Scale bar is 2 μm. (**F**) Quantification of the number of cells with a wild type (WT) phenotype (two new MACs and two new MICs) in the same experiment as in E. More than 100 cells were counted in each condition.

To examine the role of CenH3 proteins, each CenH3 protein was depleted separately during vegetative growth by feeding *P. tetraurelia* cells on double-stranded RNA-producing bacteria to induce RNA interference (24) (Figure 2A). We first checked the efficiency of protein depletion. Cells transformed with the *CENH3b-GFP* fusion construct were submitted to *CENH3a*, *CENH3b* or control RNAi, against the nonessential gene *ND7*. After 48 h, indirect immunostaining with the CenH3a antibody revealed no signal in 76% of cells subjected to *CENH3a* RNAi, indicating efficient CenH3a protein depletion (Supplementary Figure S4D). Furthermore, *CENH3a* RNAi is highly specific because 100% of CenH3a-depleted cells displayed GFP fluorescence from the CenH3b-GFP fusion protein, as in control RNAi (Figure 2B and Supplementary Figure S4D). Conversely, the number of *CENH3b-GFP* transformants displaying GFP fluorescence was greatly diminished upon *CENH3b* RNAi, while the CenH3a endogenous protein was still detected in all cells after *CENH3b* or control RNAi (Figure 2B). Quantification of the GFP signal intensity indicated approximately 75% efficacy for *CENH3b* RNAi (Supplementary Figure S4E). In conclusion, these data showed that RNAi is efficient and specific for each *CENH3* gene.

Depletion of the centromeric histone H3 protein is known to cause chromosome missegregation, cell cycle arrest and eventually cell death (37–40). To test the requirement of the CenH3 proteins in chromosome segregation, we examined the effects of CenH3a or CenH3b depletion during vegetative growth. In contrast to other organisms, no cell cycle arrest or cell death was observed upon CenH3 depletion in *Paramecium*, even after 13 divisions (Supplementary Figure S4F). This absence of proliferation phenotype is fully consistent with the fact that *Paramecium* lacking MICs do not cease dividing (36), most likely because the MICs are transcriptionally silent during vegetative growth. To reveal possible defects that could accumulate when CenH3 proteins are depleted, we looked to see whether division of the MICs was affected. Given the small size of the MIC chromosomes, we could not detect lagging MIC chromosomes. As missegregation of chromosomes may cause chromosome loss and gain that lead to aneuploidy, we quantified the number of MICs per cell upon CenH3 depletion. Following 48 h of CenH3 depletion, RNAi was stopped and cells were grown in standard food medium without dsRNA to resume normal protein expression (Figure 2A). We performed immunostaining experiments with the CenH3a antibody to count the number of MICs in each cell 48 h after RNAi release (Figure 2C). In control RNAi, 98% of cells have one to three MICs and the majority (60%) contains two MICs per cell. Similar results were obtained when cells were subjected to *CENH3b* RNAi. In contrast, depletion of CenH3a led to dramatic changes in the number of MICs per cell. Only 35% of cells showed a normal pair of MICs. Fifteen percent of the cells had aberrant numbers of nuclei with either no MIC at all or more than three MICs per cell. We conclude that CenH3a, but not CenH3b, is required for the proper division of the germline MIC during vegetative growth.

As mentioned previously, the MICs are transcriptionally silent during vegetative growth. Other defects that arise from CenH3 depletion might become obvious in the cell only when the MICs are functional. The role of the germline MICs is to ensure the faithful transmission of genetic material to the next sexual generation. We could thus check the functionality of the MIC by measuring the production of viable progeny after sexual events. A population of cells, treated with *CENH3a* or *CENH3b* RNAi for 48 h during vegetative growth then transferred to standard medium without RNAi for an additional 48 h, was then starved to trigger sexual events (Figure 2A). During the sexual process of autogamy, a self-fertilization process, the maternal MAC is destroyed and new macro- and MIC arise by meiosis, fertilization and postzygotic division of the MIC. After 3 days of starvation, individual autogamous cells were transferred to standard growth medium and allowed to resume vegetative growth and viability of sexual progeny was scored. The progeny of cells depleted for CenH3b during vegetative growth exhibited the same survival rates as the control RNAi cells, or cells that underwent autogamy in standard medium (Figure 2D). In contrast, RNAi against *CENH3a* during vegetative divisions yielded no viable sexual progeny (Figure 2D), and autogamous cells died before or at the first cell division. We conclude that CenH3a depletion abolishes the functionality of the MIC, even for cells that appeared phenotypically wild type with two MICs per cell.

CenH3a depletion during vegetative divisions led to severe developmental phenotypes. In control and *CENH3b* RNAi, autogamous cells harbored two MICs and two MACs that are easy to recognize as they progressively enlarge during development. In sharp contrast, there was a complete absence of detectable new MICs and new MACs upon CenH3a depletion (Figure 2E and F). Thus, CenH3a, but not CenH3b, is essential for the function of the germline MIC during the sexual cycle. Taken together the bioinformatic criteria, the behavior of the staining and the essential role of CenH3a strongly argue that CenH3a is the centromeric H3 in *Paramecium*.

### CenH3 loss occurs during development of the somatic MAC

**Figure 3.**
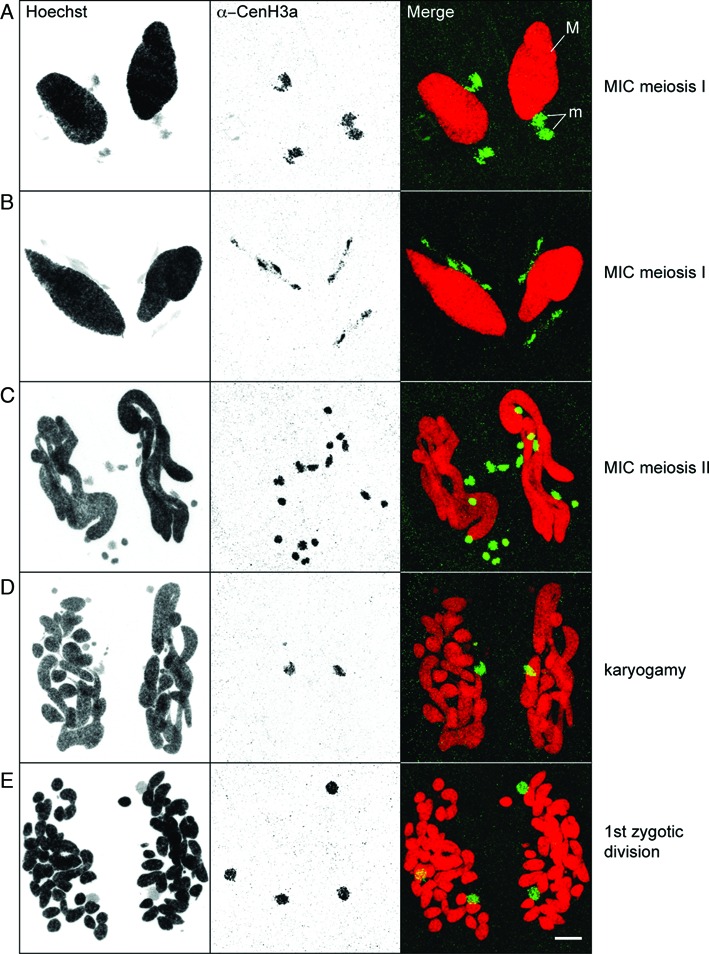
Localization of CenH3a during sexual events. Immunostaining with CenH3a antibody at the indicated stages of conjugation. M: MAC, m: MICs. Scale bar is 10 μm.

**Figure 4.**
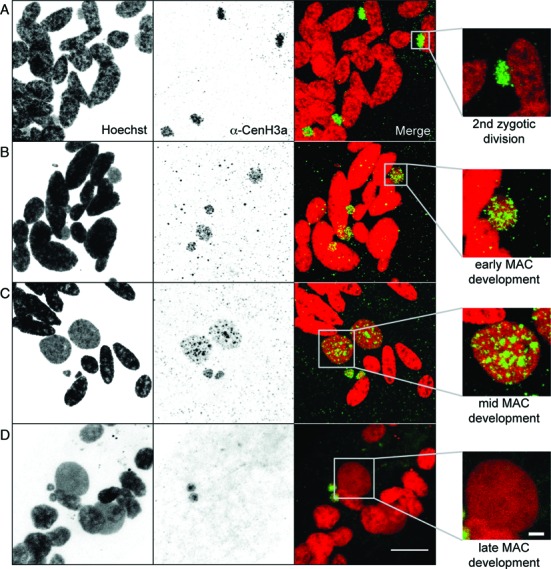
Localization of CenH3a during postzygotic events. Immunostaining with CenH3a antibody at the indicated postzygotic stages of self-fertilization process (autogamy). Scale bar is 10 μm. Magnified views of one new developing MAC are shown on the right. Scale bar is 2 μm. See also Supplementary Figure S5.

The two kinds of nuclei, MAC and MIC, have distinct centromeric activities and yet have a common origin. Whatever the mode of sexual reproduction, self-fertilization (autogamy) or cross-fertilization (conjugation), the zygotic nucleus undergoes two mitotic divisions and two of the resulting nuclei differentiate into MACs, two in MICs. To determine when distinct centromeric activities are established, we performed immunostaining experiments with the CenH3a antibody and monitored CenH3a localization during pre- and postzygotic events (Figures 3 and 4). During early conjugation, the MICs undergo meiosis and CenH3a was found in the MICs during meiosis I and meiosis II (Figure 3A–C). After completion of meiosis, one of eight haploid products is selected, while the others disintegrate. The remaining one undergoes one round of mitosis to form two haploid gametic nuclei. After reciprocal fertilization, fusion of haploid nuclei in each conjugant gives rise to the zygotic nucleus. Strikingly, we noted that CenH3a disappeared from the disintegrating nuclei while it persisted in the zygotic nucleus (Figure 3D). After the divisions of the zygotic nucleus (Figures 3E and 4A), CenH3a was found in the four diploid nuclei. At early stages of postzygotic differentiation, CenH3a was found in the two new MICs and two new MACs (Figure 4B and C). At late stages of differentiation, we noticed that the CenH3a signal concentrated in a few nuclear foci that correspond to Hoechst-poor regions (Supplementary Figure S5A). These CenH3a foci are very reminiscent of that observed for the Ezl1 protein and for Ezl1-dependent chromatin marks (H3K27me3 and H3K9me3) (19). As for Ezl1, H3K27me3 and H3K9me3, the CenH3a signal eventually disappeared within the developing MAC at later stages (Figure 4D). The new MICs, however, retained a strong CenH3a signal (Figure 4D). Loss of CenH3 in the nuclei destined to become MACs was observed for the CenH3-GFP fusion proteins as well (Supplementary Figure S5B). We conclude that centromeric protein loss occurs during development of the new somatic MAC.

### CenH3 loss is concomitant with DNA elimination events

**Figure 5.**
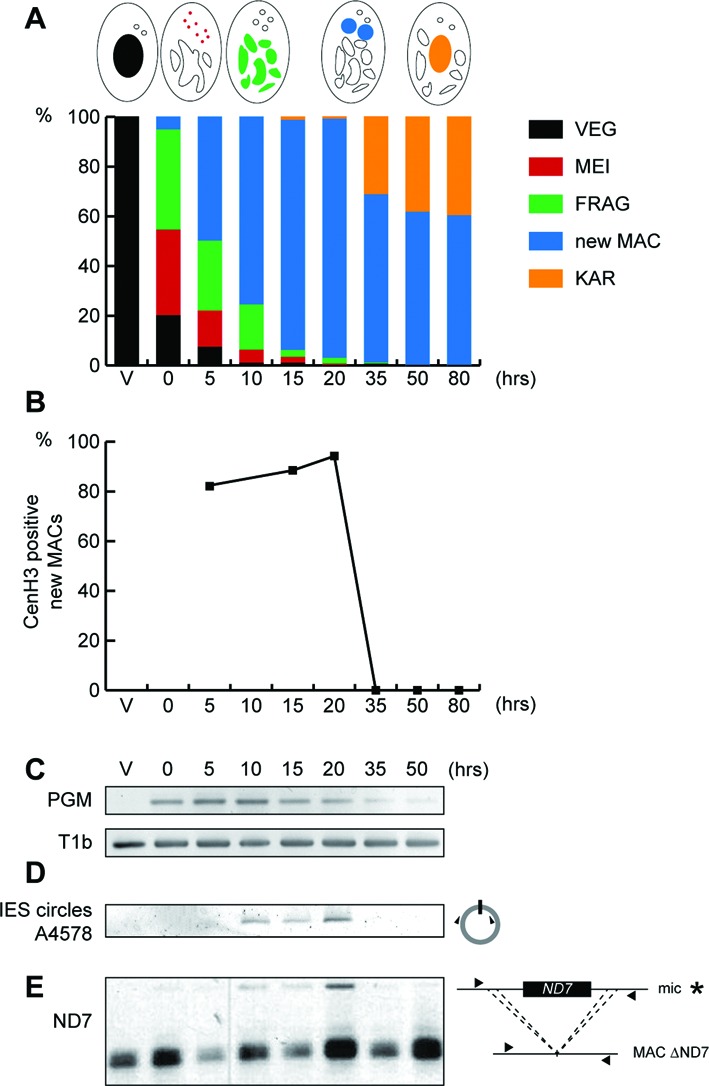
Timing of CenH3 loss coincides with that of DNA elimination. (**A**) Progression of the self-fertilization process (autogamy) was followed by cytology with Hoechst staining during a time course experiment described in (19), in which a control gene (*ICL7*) has been silenced by RNAi. Schematic representations of key nuclear events are depicted above the histogram. The time-points refer to hours after *T* = 0 h that is defined as the time when approximately 50% of cells show fragmentation of the maternal MAC (FRAG). VEG: vegetative, MEI: meiosis, new MAC: two visible new developing MACs, KAR: karyonide (first cell division). (**B**) Quantification of CenH3a positive new developing MACs was scored for at least 100 cells, at each time point of the experiment described in (A), after immunolabeling with CenH3a antibody. (**C**) Detection of *PGM* mRNA by RT-PCR. Total RNAs were extracted at each time point of the experiment described in (A), and reverse transcribed. cDNAs were amplified by PCR with gene specific primers (Supplementary Table S2) and, as a loading control, with primers for the *T1b* gene, which encodes a component of the secretory granules. (**D**) PCR detection of IES 51A4578 circles with divergent primers (triangles, Supplementary Table S2) on genomic DNA at each time point of the experiment described in (A). (**E**) Somatic deletion of the *ND7* gene. PCR analysis was performed on the same DNA samples as in (D) with primers (black arrows, Supplementary Table S2) located upstream and downstream of the *ND7* open reading frame. The faint upper band (*) corresponds to the full length MIC version of the ND7 gene, which is transiently amplified before it is deleted from the new developing MACs. The more intense lower band corresponds to rearranged forms, originating from both the maternal and new MACs.

The next step was to decipher the mechanisms that cause loss of the CenH3a protein. To define the timing of CenH3 loss, we performed an autogamy time course experiment and monitored, at each time point, the proportion of cells displaying a CenH3a signal within the new developing MACs by immunostaining. As soon as the new MACs could be distinguished from the fragments of the maternal MAC by Hoechst staining (Figure 5A), a CenH3a signal was detected within the MAC (Figure 5B). It persisted during MAC development (95% at T = 20 h). At T = 35 h, the CenH3a signal could no longer be detected within the new MACs (Figure 5B). The fact that CenH3a signal disappeared in cells containing two new MACs indicates that loss of CenH3a signal precedes the first cell division, when each new MAC segregates in daughter cells. We conclude that loss of CenH3a signal very likely involves an active mechanism that occurs independently of cell division.

The development of the new MACs involves endoreplication of the originally diploid germline genome and massive and reproducible DNA elimination (15). During this process, around 30 Mb of germline-specific sequences are removed (17). To gain further insight into the timing of CenH3 loss relative to programmed DNA elimination, we performed a molecular analysis of DNA elimination in the same autogamy time course. We first followed the expression of Pgm, the putative endonuclease required for the elimination of germline-specific sequences (18). RT-PCR analysis showed that transcription of *PGM* is switched on at early time points, the levels of *PGM* mRNA reached a peak between *T* = 5 and *T* = 10 h, before they slowly decreased at later time points during autogamy, as expected (Figure 5C). Following Pgm-dependent DNA cleavages at each IES boundary, excised linear IESs may form covalently closed circles (41). Using divergent primers internal to IES 51A4578, a 882-bp long IES from the A51 surface antigen gene (42), we monitored the appearance of excised IES circular molecules during autogamy, as shown in Figure 5D. Consistent with the expression of *PGM*, IES circles were detected starting from *T* = 10 h and accumulated during development of the new MAC. Strikingly, IES circles disappeared entirely by *T* = 35 h, the same time point as the disappearance of the CenH3a signal. To extend our analysis to another DNA elimination event, we focused on the imprecise elimination mechanism responsible for maternally inherited deletions of nonessential cellular genes. The strain used in this experiment (51deltaND7) harbors a wild type germline MIC genome, but carries a somatic MAC deletion of the nonessential gene *ND7* (43). PCR amplification allowed the detection of the *ND7* gene during development of the new MAC starting from T = 10 h. The ND7 gene was barely detectable by T = 35 h as a result of *ND7* gene deletion from the new developing MAC (Figure 5E), the same time point as the disappearance of the CenH3a signal and IES circles. Taken together, our data indicate that loss of the CenH3a protein occurs in the same developmental time window as the degradation of IES excision products and the imprecise DNA elimination events.

### Developmental loss of CenH3 requires genes involved in DNA elimination

**Figure 6.**
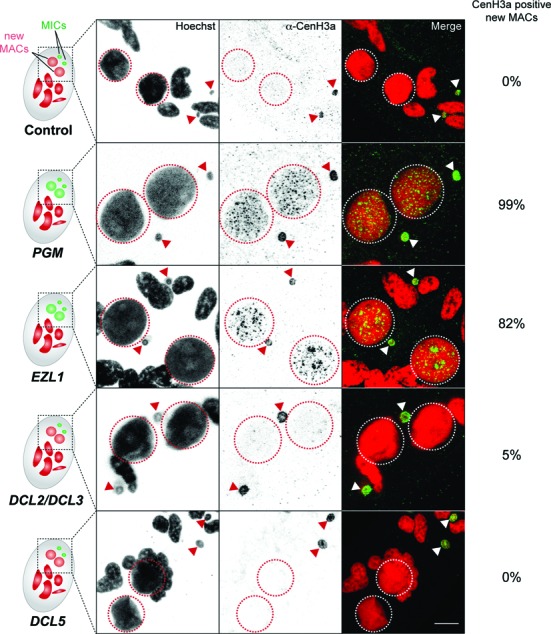
Factors involved in CenH3 loss. Immunostaining with CenH3a antibody at late stages of MAC development following control (*Paramecium* fed with *E. coli* producing dsRNAs corresponding to the plasmid L4440 with no sequence target in the *Paramecium* genome), *PGM*, *EZL1*, *DCL2* and *DCL3* or *DCL5* RNAi. Schematic representations of cells are presented on the left. Dashed circles indicate the two new developing MACs and filled arrows indicate the two MICs. Scale bar is 10 μm. Quantification of the number of cells with CenH3a positive signal in the new developing MAC was performed for at least 100 cells for each RNAi condition in two or three independent experiments. Early stages of MAC development from the same experiments are presented on Supplementary Figure S6.

Based on the above observations, it appeared that CenH3 loss might result from the physical elimination of DNA sequences associated with CenH3 from germline-derived chromosomes through the process of programmed DNA elimination. Because the Pgm endonuclease is necessary for all DNA elimination events (17), we first tested its role in the developmentally programmed loss of CenH3. Depletion of Pgm by RNAi was performed during autogamy and led to inhibition of DNA elimination and high rates of lethality in the sexual progeny (Supplementary Figure S6A and Table S1), as previously shown (18). CenH3a loss was monitored by immunostaining during development of the new MACs. At early stages of development, CenH3a was found in the new MACs and new MICs in control as well as in Pgm-depleted cells, indicating that depletion of Pgm did not prevent expression and proper nuclear localization of CenH3a (Supplementary Figure S6B). At later stages of development, the CenH3a signal was completely absent from the MAC in control cells, whereas it was still present within the developing MAC of Pgm-depleted cells, demonstrating that CenH3a disappearance requires the Pgm endonuclease (Figure 6 and Supplementary Figure S6C).

The putative histone methyltransferase Ezl1 is an essential player in the DNA elimination pathway (19). We therefore monitored CenH3a loss by immunostaining after Ezl1 depletion during autogamy. As expected, Ezl1 depletion led to inhibition of DNA elimination and high rates of lethality in the sexual progeny (Supplementary Figure S6A and Table S1). We observed that CenH3a was normally found in the new MICs and new MACs at early stages of development (Supplementary Figure S6B) but, just as for Pgm depletion, it was abnormally retained in the MACs at late stages of development (Figure 6).

To further dissect the molecular mechanisms involved in CenH3 loss, we tested the role of the scnRNAs and the iesRNAs, two distinct classes of small RNAs known to regulate a subset of DNA elimination events (19). Codepletion of Dcl2 and Dcl3 proteins, which prevents scnRNA accumulation (20,21), led to DNA elimination defects and lethality, as expected (Supplementary Figure S6A and Table S1). Yet CenH3a disappearance was not affected within the developing MACs of Dcl2/Dcl3-codepleted cells (Figure 6). Similarly, Dcl5 depletion, which impairs iesRNA accumulation (21), led to partial retention of a subset of IESs, as expected (Supplementary Figure S6A), but did not affect CenH3a disappearance within the developing MAC (Figure 6). Taken together, our data show that Pgm and Ezl1, but not Dcl2/3 or Dcl5 proteins, are needed for the developmentally programmed loss of CenH3a in the new MACs. We conclude that CenH3 loss involves the Pgm- and Ezl1-dependent elimination of germline DNA sequences, but is independent of small RNAs.

## DISCUSSION

We have identified the *Paramecium tetraurelia* centromeric protein CenH3a. We have shown its exclusive localization in the mitotically dividing germline MICs at all stages of the life cycle and its absence from the somatic MAC. Consistent with its sole localization in the MICs, depletion of the CenH3a protein by RNAi causes division defects of the MICs but does not appear to affect MAC division. These observations strongly support the idea that MAC chromosomes do not harbor active centromeres. Absence of CenH3 proteins is a general feature of terminally differentiated cells or senescent cells (44–47), which, in contrast to the somatic MAC, no longer divide.

Loss of the CenH3a protein is part of a developmental program that leads to the formation of the new somatic MAC after sexual events. Moreover, the timing of CenH3a loss coincides with programmed DNA elimination events, which occur in the new developing MAC as well. We ruled out that the *CENH3a* gene itself is deleted through developmentally programmed DNA elimination, because the gene is present in the MAC genome assembly (25,26). To understand the mechanism for CenH3 loss, we carried out a gene candidate approach by RNAi testing genes involved in programmed DNA elimination. Two proteins whose depletion led to retention of the CenH3a protein in the somatic MAC were identified: the domesticated transposase Pgm and the Polycomb-like histone methyltransferase Ezl1. In contrast, both codepletion of the Dicer-like proteins, Dcl2 and Dcl3, and depletion of Dcl5, involved in the biogenesis of scnRNAs and iesRNAs respectively, did not affect CenH3a removal during MAC development, although some DNA elimination was impaired. High-throughput sequencing indicated that only a small fraction of IESs, less than 10%, are retained in the new MACs after codepletion of Dcl2 and Dcl3, or depletion of Dcl5, whereas all IESs are retained after Pgm depletion and 70% after Ezl1 depletion (19). Thus, the CenH3a protein, like the vast majority of IESs, is correctly removed from the new developing MAC in the complete absence of scnRNAs or iesRNAs.

Analysis of high-throughput sequencing of polyadenylated RNAs extracted at different time points during autogamy showed that *CENH3a* RNA levels are not affected upon Pgm or Ezl1 depletion as compared to control (O. Arnaiz, personal communication). Thus, retention of the CenH3a protein in the developing MAC after Pgm and Ezl1 depletion is not due to an increase of *CENH3a* mRNA levels. We think the simplest hypothesis to explain our observations is that loss of CenH3a protein reflects loss of CenH3-associated DNA. We propose a two-step scenario: (i) excision from the germline chromosomes of the centromere DNA through the action of the Pgm endonuclease and the Ezl1 histone methyltransferase, followed by (ii) degradation of the centromere DNA and associated CenH3 proteins. We hypothesize that the role of the Ezl1 protein in CenH3 loss is to trimethylate lysine 27 and lysine 9 on centromere-associated H3 nucleosomes, which are generally interspersed with CenH3 nucleosomes (48). Both histone marks are found in the new MACs at the time DNA elimination events occur and, as observed for CenH3 proteins, concentrate in a few nuclear foci then disappear from the late developing MAC (19). The Pgm endonuclease could be guided to its DNA cleavage sites through the reading of these chromatin modifications via its PHD-like domain, as proposed for other germline eliminated DNA segments (49). Once excised, extra-chromosomal CenH3-associated DNA, like IES circles, would be degraded. Interestingly, nondividing *Arabidopsis* pollen vegetative cells also undergo loss of CenH3, as part of a developmental program (44,47). This removal of CenH3 involves the CDC48A AAA-ATPase molecular chaperone (50). Similarly, an active mechanism may be responsible for degradation of the CenH3-associated DNA.

Identification of the sequence of the centromeres would be necessary for a definitive proof that separation of centromere activities between the germline and the somatic nuclei is due to deletion of CenH3-associated DNA. It is currently technically not possible but ongoing studies of the MIC genome will be instrumental to determine the sequence and annotation of the centromeres. If centromeres are indeed deleted from the MAC, it will provide the first evidence that eliminated DNA has an essential biological function in the germline. It may also explain the selective pressure to maintain sequences in the germline genome that are readily removed from the somatic genome.

How the MAC divides in the absence of CenH3 remains a mystery (51,52). There are several documented cases of nuclear division in the absence of CenH3. In multiple insect lineages that have lost CenH3 during evolution, mitosis relies on an uncharacterized CenH3-independent mechanism (53). In kinetoplastids, the absence of conventional centromeric proteins is compensated by a set of specific kinetochore components (54). Ciliated protozoan innovated a different strategy, which conferred to the polyploid somatic MAC the unique ability to carry out a nonmitotic division. There does not appear to be any mechanism to ensure equal segregation of duplicated MAC molecules to the two daughter cells. Instead, it is likely that the high ploidy level of the MAC (∼800n) in *P. tetraurelia* prevents lethal gene loss for a number of vegetative divisions.

Altogether, our work shows that *Paramecium*, in which centromere loss is inducible and reproducible, provides an excellent model organism to dissect the mechanisms involved in this process. Our results strongly argue that the mechanism of centromere loss involves deletion of DNA upon which CenH3 is assembled. We demonstrate the essential roles for an endonuclease and a histone methyltransferase in DNA deletion-mediated centromere loss. Future work will show whether this represents a universal mechanism used by other organisms.

## SUPPLEMENTARY DATA

Supplementary Data are available at NAR Online.

SUPPLEMENTARY DATA
