# Supplementary material for: DNA deletion as a mechanism for developmentally programmed centromere loss
Source: Nucleic Acids Res. 2015 Oct 25;44(4):1553–65. doi: 10.1093/nar/gkv1110 (PMC4770206; doi:10.1093/nar/gkv1110)
Supplement: SUPPLEMENTARY DATA [file supp_44_4_1553__index.html]

DNA deletion as a mechanism for developmentally programmed centromere loss — SUPPLEMENTARY DATA 

# DNA deletion as a mechanism for developmentally programmed centromere loss

## SUPPLEMENTARY DATA

- SUPPLEMENTARY DATA
