# Supplementary material for: DNA deletion as a mechanism for developmentally programmed centromere loss
Source: Nucleic Acids Res. 2015 Oct 25;44(4):1553–65. doi: 10.1093/nar/gkv1110 (PMC4770206; doi:10.1093/nar/gkv1110)
Supplement: SUPPLEMENTARY DATA [file supp_gkv1110_Lhuillier_SupplementaryData.pdf]

Supplementary Data

DNA deletion as a mechanism for developmentally programmed centromere loss

Maoussi Lhuillier-Akakpo, Frédéric Guérin, Andrea Frapporti and Sandra Duharcourt

Supplementary Figures S1-S6
Supplementary Tables S1-S2
Supplementary References

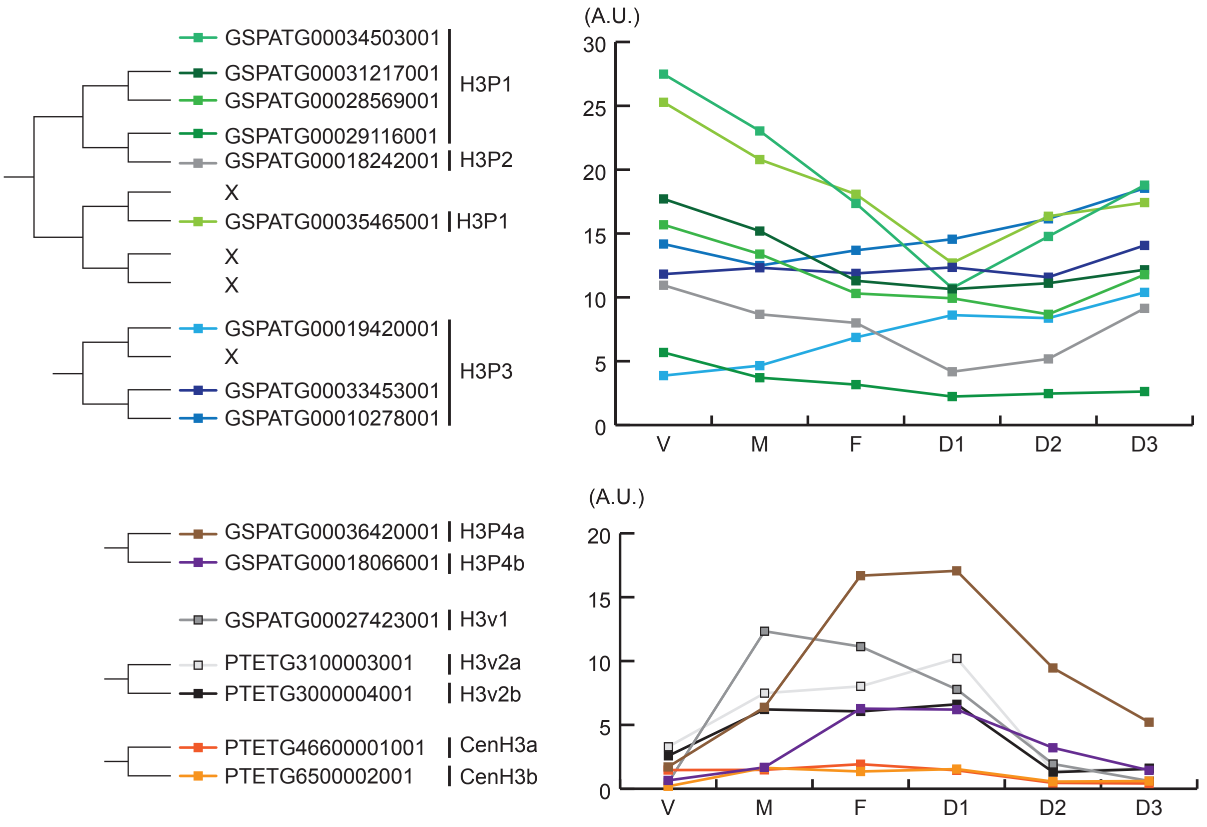

Figure S1, Related to Figure 1. Gene expression levels of H3 variants
Diagram of the whole genome duplication relationships between H3 and H3 variant genes in *P. tetraurelia*. Gene accession numbers and the corresponding encoded proteins are indicated. Gene expression profile of H3 genes from vegetative culture (V) and different stages of development. Gene expression levels in arbitrary units (in thousands) are from a previous study (58). According to (58), the five developmental stages following vegetative growth (V) are: (M) cells undergoing meiosis, at the beginning of MAC fragmentation; (F) 50% of cells with fragmented maternal MAC; corresponding to our T=0hr (Figure 5); (D1) significant proportion of cells with visible new MACs; corresponding to our T=5hrs (Figure 5); (D2) the majority of cells have a detectable new MAC, corresponding to our T=10hrs; (D3) population of cells 10 hr after D2, corresponding to our T=20hrs. The H3P4 (putative H3.3, see Figure S2) genes are specifically induced during the development of the new MAC. In contrast, most other canonical H3 genes are expressed at high levels throughout the life cycle. Interestingly, the gene encoding the H3v1 variant is specifically induced at early stages of the sexual cycle, while the H3v2 genes are specifically induced later during development of the new MAC, as observed for the putative H3.3 variants (H3P4a-H3P4b). These developmental specific histone H3 variants might regulate the massive nuclear reorganization events that are taking place during development, as suggested in the distantly related ciliate *Euplotes crassus* (59).

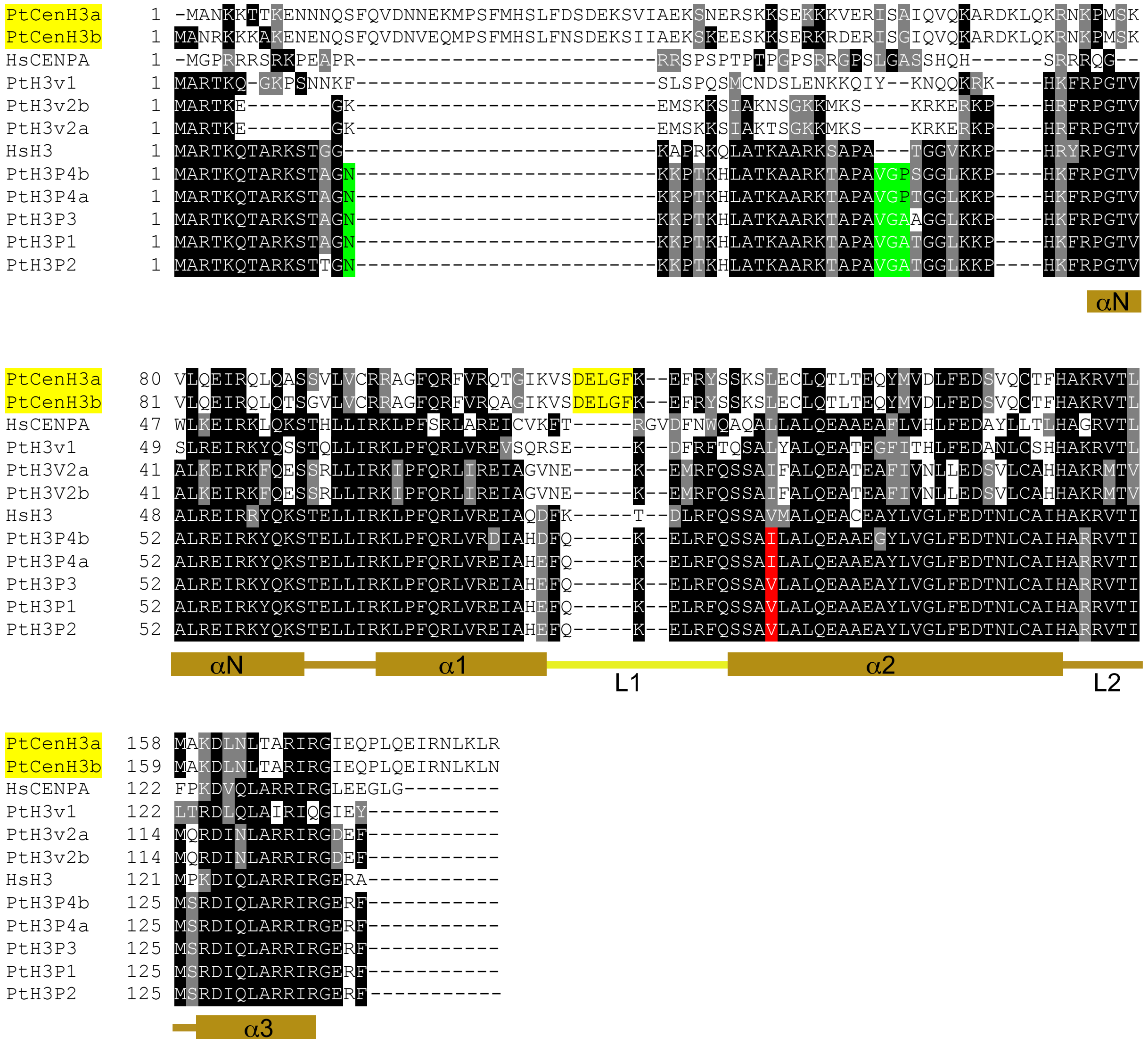

Figure S2, Related to Figure 1. H3 variants in *P. tetraurelia*
The entire protein sequences of H3 from *P. tetraurelia* (gene accession numbers are available in Figure S1) and *H. sapiens* (accession number 68431) were aligned using the MUSCLE software (56). Conserved residues are highlighted black and grey. *P. tetraurelia* CenH3 proteins (PtCenH3a, PtCenH3b) are highlighted in yellow on the left. Below the alignment is a schematic representation of the domain organization of histone H3, with the N-terminal α-helix (αN), α-helices 1-3 (α1-3) and loops 1 and 2 (L1, L2). Additional amino acids found in PtCenH3a and PtCenH3b in the first loop (L1) of the histone fold domain are highlighted in yellow. One amino acid (at position 89, highlighted in red) described to discriminate H3.1 and H3.3 (28) distinguishes H3P1-3 from H3P4a-H3P4b. Additional amino acids found in *P. tetraurelia* H3P1-3 and putative H3.3 (H3P4a-H3P4b) proteins are highlighted in green.

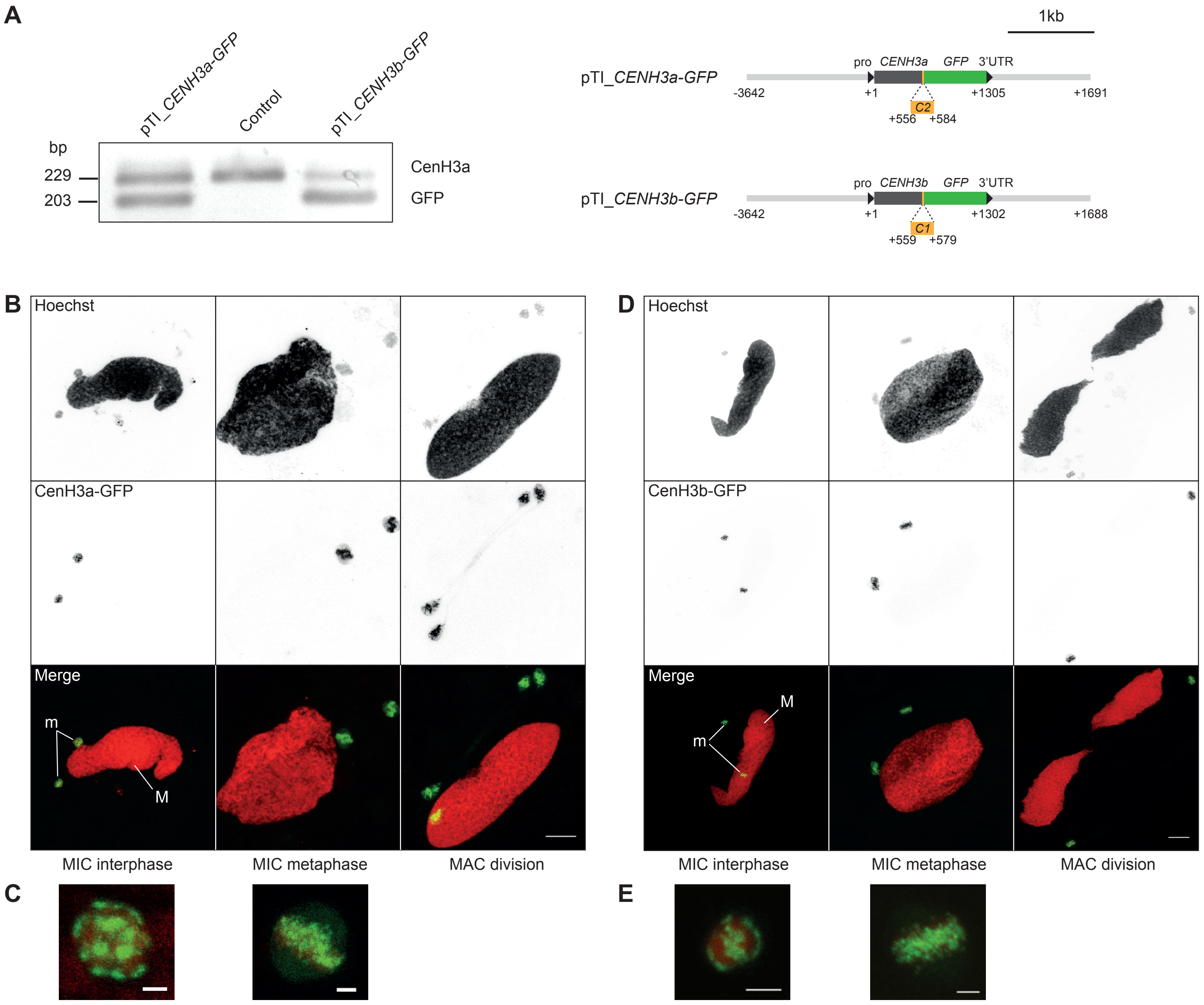

Figure S3, Related to Figure 1. Localization of CenH3a-GFP and CenH3b-GFP proteins

A. Transformation with *CENH3a-GFP* or *CENH3b-GFP* fusion transgenes. Transformed clones were tested with a duplex PCR (see Table S2): a plasmid-specific PCR amplifies a 203-pb fragment within the *GFP* gene and a gene-specific PCR amplifies a 229-bp fragment from the endogenous *CENH3a* gene. The relative abundance of the two products gives an indication of plasmid copy number. In these GFP transformants, the fusion proteins are exclusively localized to the MICs. Linearized transgene plasmid constructs are drawn on the right. In both pTI-derived plasmids, the GFP fusion proteins are constitutively expressed under the Elongation Factor Tu transcription signals (promoter and 3'UTR). Linker sequences (encoding SRPVAT (C1) or GGSGGSGGS (C2)) are located between the *CENH3* and the *GFP* coding sequences. B. Localization of CenH3a-GFP at different stages of the cell cycle. Note that CenH3a-GFP is detected on the mitotic spindle. M: MAC, m: MICs. Scale bar is 10 µm. C. Magnified views of the MICs during interphase and metaphase. Scale bar is 2 µm. D. Localization of CenH3b-GFP during the cell cycle. Only one out of two MICs is visible in each daughter cell during MAC division M: MAC, m: MICs. Scale bar is 10 µm. E. Magnified views of the MICs during interphase and metaphase. Scale bar is 2 µm.

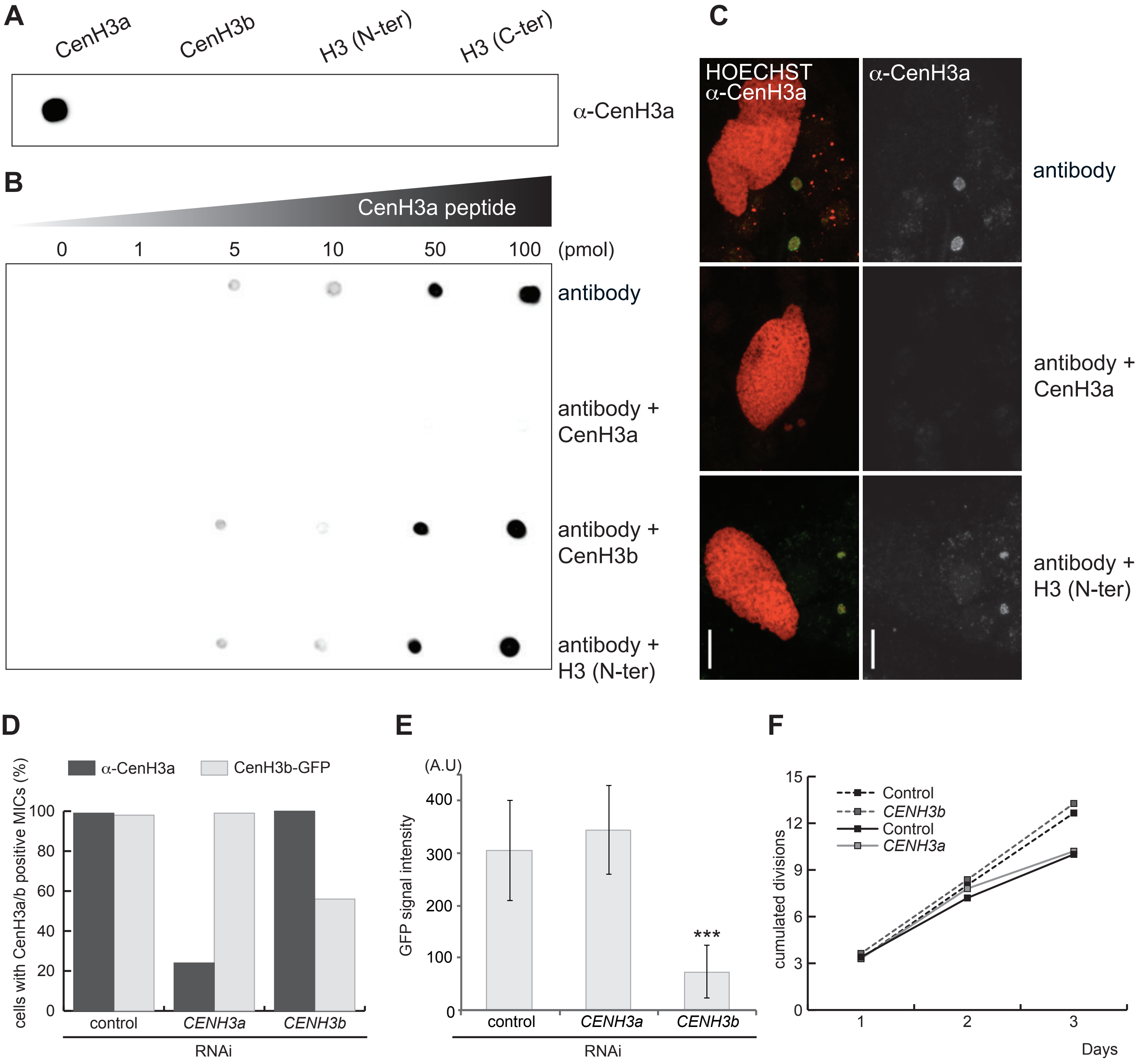

Figure S4. Specificity of CenH3a antibody. Efficiency, specificity and effect on cell proliferation of *CENH3a* and *CENH3b* RNAi.

A. Dot blot assay using *P. tetraurelia* CenH3 and H3 peptides. 100 pmol of each of the indicated peptides were spotted on the membrane and probed with the CenH3a antibodies. Polyclonal rabbit antibodies raised against CenH3a peptide showed specific reactivity with CenH3a peptide (CenH3a: KTTKENNNQSFQVDNNEKMP) but not against CenH3b (CenH3b: KKAKENENQSFQVDNVEQMP), H3 N-terminal region (H3 N-ter: TKAARKTAPC) or H3 C-terminal region (H3 C-ter, MSRDIQLARRIRGERF). CenH3a antibodies are specific for CenH3a peptide and do not cross react with CenH3b peptide. B. Competition assay (dot blot). 0 to 100 pmol of CenH3a peptide were spotted and probed with CenH3a antibodies alone or in presence of a 50-fold molar excess of the indicated peptides. Competition with the CenH3a peptide (CenH3a) completely eliminates the signal, while competition with other peptides (CenH3b, H3 N-ter) does not. C. Competition assay (immunostaining). Immunostaining was performed with CenH3a antibodies alone or in presence of a 50-fold molar excess of the indicated peptides. Competition with the CenH3a peptide (CenH3a) completely eliminates the signal ((0/114) 0% cells with a signal in the MICs, while competition with the other peptide (H3 N-ter) does not ((200/200) 100% cells with a signal in the MICs in presence of the H3 N-ter peptide, as observed in the absence of peptide (antibody alone) (123/123)). Scale bar is 10 μm. D. The number of cells exhibiting CenH3a or CenH3b proteins in the MICs was scored after 48 hours of control (*ND7*), *CENH3a* or *CENH3b* RNAi. CenH3a was detected by immunostaining with CenH3a antibody (dark grey bars) and CenH3b was detected by GFP fluorescence in *CENH3b-GFP* transformed cells (light grey bars). More than 100 cells were scored in each condition. E. Quantification of GFP fluorescence intensities in the MICs of *CENH3b-GFP* transformed cells (see Materials and Methods). Error bars indicate the standard deviation. *** indicate significant differences as determined by a statistical Z-test (p<10-10). F. In a population of exponentially growing cells, the number of cumulated divisions was scored for three days upon *CENH3a*, *CENH3b* or *ND7* (control) RNAi. In the *CENH3a* RNAi experiment (full lines), RNAi efficacy was assessed by the recovery of viable post-autogamous progeny (100% progeny with functional new MACs for control RNAi and 0% for *CENH3a* RNAi). In the *CENH3b* RNAi experiment (dotted lines), RNAi efficacy was assessed by GFP detection in *CENH3b-GFP* transformed cells (as in Figure S4D) (at day 2, 100% cells with GFP in the MICs for control RNAi and 48% for *CENH3b* RNAi). The efficiency of *ND7* RNAi was confirmed by the lack of trichocyst discharge in the presence of picric acid.

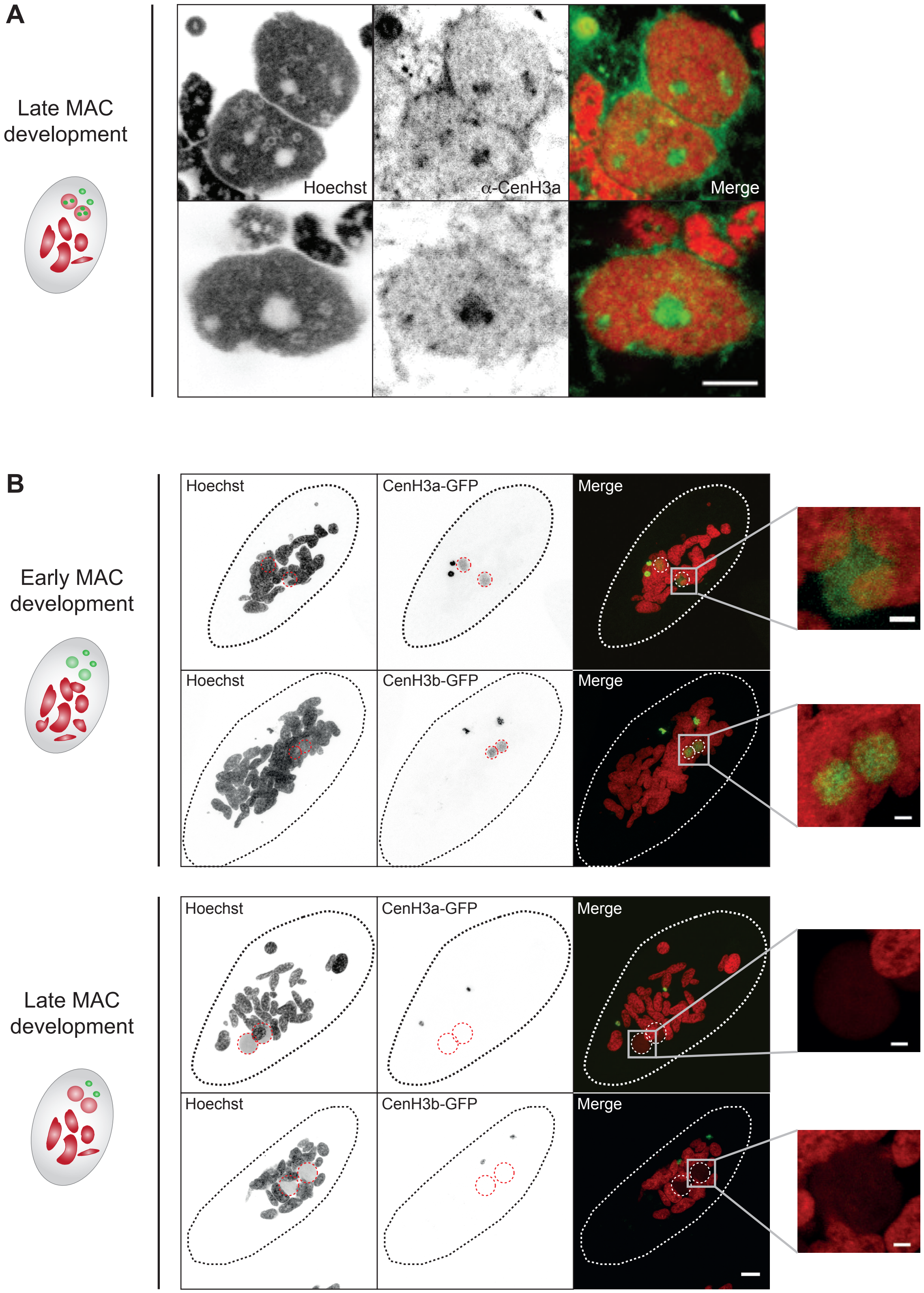

Figure S5, Related to Figure 4. Sub-nuclear localization of CenH3a at late stages of macronuclear development and localization of CenH3a-GFP and CenH3b-GFP during macronuclear development

A. Immunostaining with CenH3a antibody. Selected stacks of magnified views of developing MACs at late stages are presented. A schematic representation is shown on the left. Scale bar is 5 µm. B. Localization of CenH3a-GFP and CenH3b-GFP fusion proteins at early and late stages of MAC development. Scale bar is 10 µm. Schematic representations of cells are shown on the left. Magnified views of one new developing MAC are shown on the right. Scale bar is 2 µm.

Figure S6

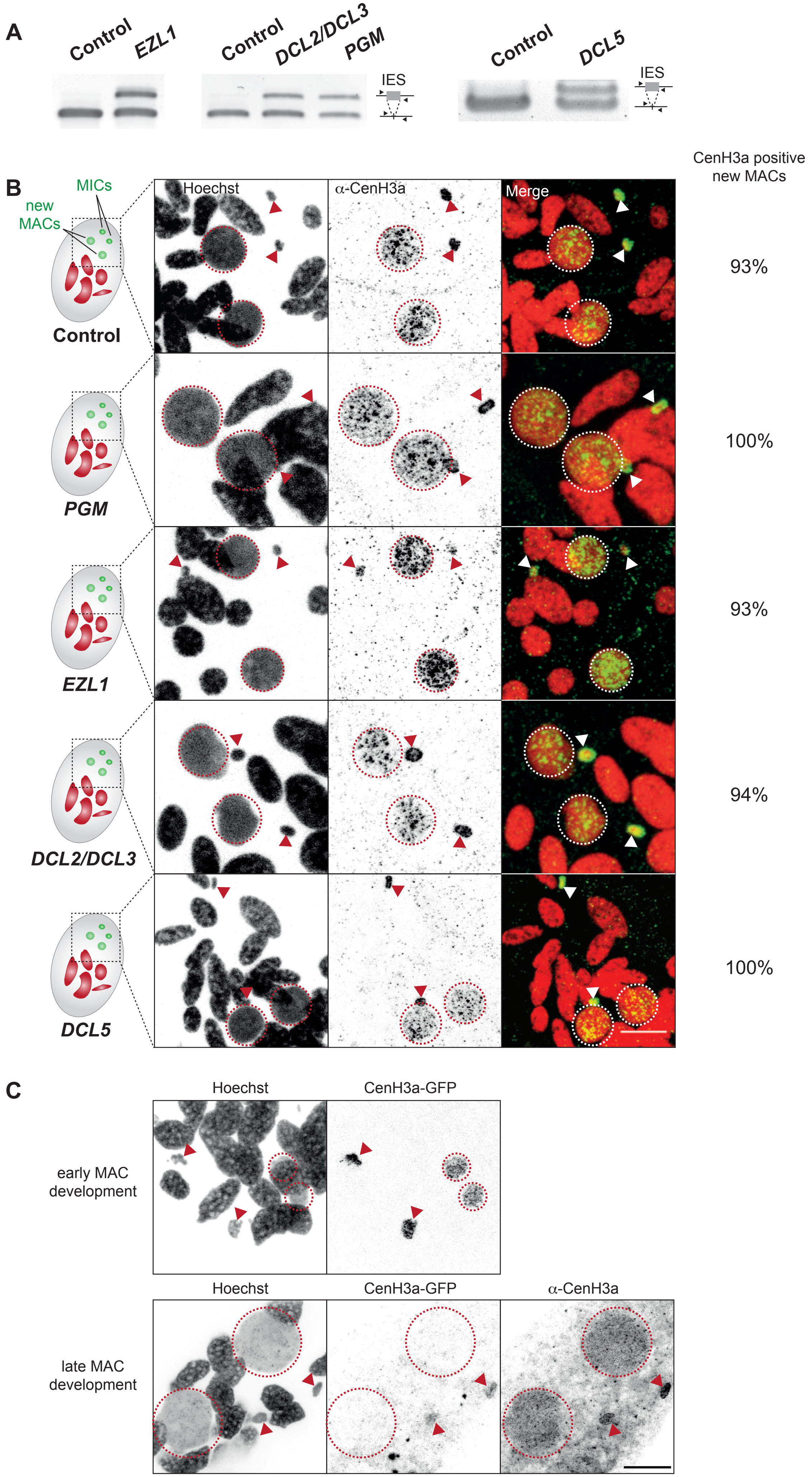
A
Control
EZL1
Control
DCL2/DCL3
PGM
IES
Control
DCL5
IES
B
MICs
new MACs
Control
PGM
EZL1
DCL2/DCL3
DCL5
Hoechst
α-CenH3a
Merge
CenH3a positive new MACs
93%
100%
93%
94%
100%
C
Hoechst
CenH3a-GFP
early MAC development
Hoechst
CenH3a-GFP
α-CenH3a
late MAC development

Figure S6. Related to Figure 6. Factors involved in CenH3 loss

A. PCR analysis of IES retention after *EZL1, DCL2* and *DCL3, PGM*, or *DCL5* RNAi. PCR analysis of IES retention with primers (black arrows, Table S2) located on either side of the 185-bp mtA IES (60) or of a 27-bp IES (21) in mass autogamies after RNAi-mediated silencing of the indicated genes. Total DNA samples were prepared from starved post-autogamous cells at approximately 72 hrs from the experiments presented in panel B. Because the maternal MAC is still present at this stage, the excised version is amplified in all cases; the IES-retaining fragment can be detected only if it accumulates in the zygotic developing MACs. Control: unrelated negative control (*ND7* RNAi or *Paramecium* fed with *E. coli* producing dsRNAs corresponding to the plasmid L4440 with no sequence target in the *Paramecium* genome). B. Immunostaining with CenH3a antibody at early stages of MAC development from control, *PGM, EZL1, DCL2* and *DCL3* or *DCL5* RNAi from the experiments shown in Figure 6. Schematic representations of cells are presented on the left. Dashed circles indicate the two new developing MACs and filled arrows indicate the two MICs. Scale bar is 10 µm. Quantification of the number of cells with CenH3a positive signal in the new developing MACs was performed for at least 100 cells for each RNAi condition in three independent experiments. C. Immunostaining with CenH3a antibody of *CENH3a-GFP* transformed cells during MAC development following *PGM* RNAi. Scale bar is 10 µm. The *CENH3a-GFP* transgene only allows detection of expression from the maternal macronucleus. The presence of the CenH3a-GFP fusion protein in the early developing MAC (see also Figure S5B) indicates that maternal CenH3 is inherited by the zygote and its mitotic products. The endogenous CenH3a protein, revealed with the CenH3a antibody, is retained in Pgm-depleted cells at late stages of development, whereas the CenH3a-GFP fusion protein is not. The presence of only the endogenous CenH3a protein in the new developing MACs indicates that the GFP fusion protein has a short half-life or that *de novo* synthesis of zygotic CenH3a begins early during nuclear differentiation.

Table S1. Recovery of viable post-autogamous progeny after RNAi-mediated silencing of the indicated genes

	Control[1]	*PGM*	*EZL1*	*DCL2-DCL3*	*DCL5*
Experiment 1	96% (ND7)	12%	16%	29%	-
Experiment 2	94% (L4440)	29%	-	23%	-
Experiment 3	90% (L4440)	-	0%	-	-
Experiment 4	90% (ND7)	0%	0%	4%	-
Experiment 5	100% (ND7)	-	-	-	100%
Experiment 6	100% (ND7)	-	-	-	100%

[1]As indicated in parenthesis, control correspond to *ND7* RNAi or *Paramecium* fed with *E. coli* producing dsRNAs corresponding to the plasmid L4440 with no sequence target in the *Paramecium* genome. Survival of the progeny was tested by transferring 30 to 48 individual autogamous cells to standard *K. pneumoniae* medium.

Table S2. Oligonucleotides used in this study

Name	Sequence (5′ to 3′)	Application
KB41	TGAAGGTGATGCAACTTATGGAAAATTAAC	Transgene copy number
KB42	TGAAGAATATAGTTCTTTCTTGGACATATC	Transgene copy number
CENH3aF	TGCTAATAAAAAGACGACTAAAGAGAATAATAAC	Transgene copy number
CENH3aR	CATCGGTTTATTTCTCTTTTAAAG	Transgene copy number
bigND7_5'	GATATACGTAAAATGATTGAATTGGTTTAC	PCR around ND7 gene
bigND7_3'	TTCATAAGTTAACTTCTTAACTGATAATGC	PCR around ND7 gene
PGM_F2	GAAAGAGGTACAGGAATGTGAATTC	RT-PCR PGM
PGM_R2	GCAGTTTCGGATCATCAAACAAGGATC	RT-PCR PGM
T1b_5'(2)b	TCTAATTAAACCAAGAACACGCTGAATTC	RT-PCR T1b
T1b_3'	TTGAGTTGGGATTTGACATAATCGGTGAA	RT-PCR T1b
51A4578-2	TGGTTGTTAGTCTCAAAGAATTCTAAAGAC	PCR for IES circles
514578-7bis	AAATTCCAAAAAGTTTTGAATATCTTTTGAG	PCR for IES circles
mtAF6	GGTGTTTATATCTTAATTGTTGACCCCTCAC	PCR around IES mtA
mtAR7	CCATCTATACTCCATTCTTTATCTTAATTCAT	PCR around IES mtA
IES3_DCL5_1	ATTTAACTTCCAACTCCATCTTTCATTG	PCR around IES
IES3_DCL5_2	TACCATAGTTAGAAAAATTAAGTAGTTC	PCR around IES

Supplementary References

58. Arnaiz,O., Gout,J.F., Betermier,M., Bouhouche,K., Cohen,J., Duret,L., Kapusta,A., Meyer,E. and Sperling,L. (2010) Gene expression in a paleopolyploid: a transcriptome resource for the ciliate Paramecium tetraurelia. *BMC Genomics*, 11, 547.

59. Jahn,C.L., Ling,Z., Tebeau,C.M. and Klobutcher,L.A. (1997) An unusual histone H3 specific for early macronuclear development in Euplotes crassus. *Proc. Natl. Acad. Sci. U.S.A.*, 94, 1332–1337.

60. Singh,D.P., Saudemont,B., Guglielmi,G., Arnaiz,O., Goût,J.-F., Prajer,M., Potekhin,A., Przybos,E., Aubusson-Fleury,A., Bhullar,S. et al. (2014) Genome-defence small RNAs exapted for epigenetic mating-type inheritance. *Nature*, 509, 447–452.
